# Towards a unified open access dataset of molecular interactions

**DOI:** 10.1038/s41467-020-19942-z

**Published:** 2020-12-01

**Authors:** Pablo Porras, Elisabet Barrera, Alan Bridge, Noemi del-Toro, Gianni Cesareni, Margaret Duesbury, Henning Hermjakob, Marta Iannuccelli, Igor Jurisica, Max Kotlyar, Luana Licata, Ruth C. Lovering, David J. Lynn, Birgit Meldal, Bindu Nanduri, Kalpana Paneerselvam, Simona Panni, Chiara Pastrello, Matteo Pellegrini, Livia Perfetto, Negin Rahimzadeh, Prashansa Ratan, Sylvie Ricard-Blum, Lukasz Salwinski, Gautam Shirodkar, Anjalia Shrivastava, Sandra Orchard

**Affiliations:** 1 European Molecular Biology Laboratory, European Bioinformatics Institute (EMBL-EBI), Wellcome Trust Campus, Hinxton, Cambridge, CB10 1SD UK; 2 SIB Swiss Institute of Bioinformatics, Centre Medical Universitaire, 1 rue Michel Servet, CH-1211 Geneva, Switzerland; 3 University of Rome Tor Vergata, Rome, Italy; 4 IRCCS Fondazione Santa Lucia, 00143 Rome, Italy; 5 UCLA-DOE Institute, University of California, Los Angeles, CA 90095 USA; 6 Osteoarthritis Research Program, Division of Orthopedic Surgery, Schroeder Arthritis Institute, and Krembil Research Institute, University Health Network, 60 Leonard Avenue, 5KD-407, Toronto, ON M5T 0S8 Canada; 7 Departments of Medical Biophysics, and Computer Science, University of Toronto, Toronto, ON Canada; 8 Institute of Neuroimmunology, Slovak Academy of Sciences, Bratislava, Slovakia; 9 Functional Gene Annotation, Preclinical and Fundamental Science, UCL Institute of Cardiovascular Science, University College London, London, WC1E 6JF UK; 10 Computational and Systems Biology Program, Precision Medicine Theme, South Australian Health and Medical Research Institute, Adelaide, SA 5000 Australia; 11 College of Medicine and Public Health, Flinders University, Bedford Park, SA 5042 Australia; 12 Institute for Genomics, Biocomputing and Biotechnology, Mississippi State University, Starkville, MS USA; 13 Università della Calabria, Dipartimento di Biologia, Ecologia e Scienze della Terra, Via Pietro Bucci Cubo 6/C, Rende, CS Italy; 14 Department of Molecular, Cell and Developmental Biology, UCLA, Box 951606, Los Angeles, CA 90095-1606 USA; 15 ICBMS, UMR 5246 University Lyon 1 - CNRS, Univ. Lyon, 69622 Villeurbanne, France; 16 Open Targets, Wellcome Genome Campus, Hinxton, Cambridge, CB10 1SD UK

**Keywords:** Protein-protein interaction networks, Data publication and archiving, Protein databases, Biochemical networks, Standardization

## Abstract

The International Molecular Exchange (IMEx) Consortium provides scientists with a single body of experimentally verified protein interactions curated in rich contextual detail to an internationally agreed standard. In this update to the work of the IMEx Consortium, we discuss how this initiative has been working in practice, how it has ensured database sustainability, and how it is meeting emerging annotation challenges through the introduction of new interactor types and data formats. Additionally, we provide examples of how IMEx data are being used by biomedical researchers and integrated in other bioinformatic tools and resources.

## Abstract (web summary)

The IMEx consortium provides one of the largest resources of curated, experimentally verified molecular interaction data. Here, the authors review how IMEx evolved into a fundamental resource for life scientists and describe how IMEx data can support biomedical research.

## Introduction

Proteins do not function in isolation, but rather operate through complex networks of interactions with other molecules. To truly understand cell signalling, it is necessary to get a handle on the temporal and spatial contexts within which molecular interactions occur, and how signals flow through the resulting dynamic molecular networks. Perturbations in this information flow often result in disease. We therefore also need to recognise how molecular interaction networks are reconfigured in disease, for example, via nonsynonymous mutations that alter the interactions of mutant proteins with other molecules.

Studying the interactome ― the set of all intermolecular interactions within a cell ― enables researchers to interrogate the functional consequences of variation, and gain insight into disease processes^1^. However, interactome descriptions currently suffer from two fundamental problems: noise (false positives) and lack of coverage (false negatives and unexplored interactome space)^2,3^. In the early days of technique development there were serious concerns about the reliability of methods such as protein complementation assays, exemplified by yeast 2-hybrid, or affinity purification techniques^4^, but these technical issues have largely been overcome. Nowadays experts in the field are more concerned with a lack of understanding of to what extent given protein–protein interactions (PPIs) are determined by the specific tissues, cell types or experimental conditions under which they are observed^3^.

Various techniques are used to identify PPIs, which often detect different subsets of the interactions potentially occurring within the same targeted interaction space^5,6^. Methodological issues may thus partly explain the frequent lack of significant overlap between large-scale PPI datasets. Therefore, in order to correctly interpret PPI data, it is important to understand the context in which the data was collected. This includes not only the experimental technique and the type of relationship it will detect (such as direct binding between two partners identified by X-ray crystallography, affinity purification of multiple proteins complexes or colocalization of two proteins in the same cellular compartment) but also the experimental conditions and modifications made to the participating molecules such as affinity tags or sequence mutations. All these metadata will impact the composition of the interactome generated. Therefore, this metadata need to be recorded in a computationally accessible manner, enabling researchers to make informed decisions as to the quality of data they are working with. The International Molecular Exchange (IMEx) Consortium^7^ was formed in 2005 with the goal to provide users with a dataset enhanced with controlled vocabulary (CV) terms to enable scoring, filtering and sophisticated searching of the information.

Here, we review the advances made in curation practices and data formats since the IMEx Consortium was first described in 2012^7^. We illustrate additional ways in which the data can be scored and filtered; and describe use cases where researchers have moved beyond simple high-coverage, gene-centric networks to use the additional level of detail provided by IMEx Consortium data in both analysis and visualisation.

## The IMEx consortium

The IMEx Consortium is open to any group or resource interested in curating physical molecular interactions, current members include IntAct^8^, MINT^8^, DIP^9^, UniProt^10^, MatrixDB^11^, InnateDB^12^, HPIDB^13^, UCL Functional Gene Annotation team and IID^14^. The consortium comprises the majority of the existing database resources who have agreed to collaborate on the curation of published, experimentally derived interaction data. The IMEx Consortium members have agreed on a set of curation rules and map interaction data to a limited set of defined molecule identifiers to provide the user with a single and consistent dataset, with each interaction being assigned a unique and persistent identifier.

While the IMEx Consortium is a global effort with contributing members from Europe, North America and Australasia, its common rigorous curation rules and standards have allowed IMEx to be selected as one of the Core Data Resources of the European Life Sciences Infrastructure for Biological Information (ELIXIR)^15^, which are considered essential for the long-term preservation of biological data. At the same time the IMEx Consortium continues to provide an enhanced service to both research funders and data users^16^.

## The IMEx data distribution model

The formation of the IMEx Consortium was a natural progression from the work of the Molecular Interaction (MI) working group of the Human Proteome Organization Proteomics Standards Initiative (HUPO-PSI). This group has developed the now widely implemented PSI-MI XML2.5 data interchange format^17^. They recently published an update to enable the description of more complex data types such as cooperative/allosteric interactions and dynamic interactions (PSI-MI XML3.0)^18^, and also produced simpler, tab-delimited representations (MITAB), which can be more rapidly parsed or downloaded. In addition to a tool suite and libraries designed to utilise these formats, HUPO-PSI maintains the associated MI CV (www.ebi.ac.uk/ols/ontologies/mi) that contains terms to describe all aspects of an interaction record. All IMEx data are made publicly available in the HUPO-PSI standard formats, making them Findable, Accessible, Interoperable and Reusable (FAIR)^19^.

The initial data distribution model consisted of interaction databases retaining their data locally and making their IMEx dataset available through the Proteomics Standard Initiative Common QUery InterfaCe (PSICQUIC)^20,21^ (see also section “TOOLS TO VISUALIZE AND QUERY IMEx DATA” below). However, even at the IMEx website, each resource’s records were listed separately and users had to cluster the results of their search to merge different evidences for the same interacting pair of molecules. Although a tool to enable this was supplied at the site, it was restricted to operating on <5000 records.

As database infrastructure funding has become more difficult to obtain, members of the IMEx Consortium agreed 3 years ago to centralise their IMEx-compliant data-storage and curation efforts in the IntAct database maintained at the EMBL-European Bioinformatics Institute (EBI). This enables resources to concentrate on the curation effort, rather than development of curation pipelines and annotation tools^16^, and also increases curation consistency. Members enter data through a web-based editorial platform designed to allow collaborative curation by physically remote partners. A sophisticated institute manager module links individual curators to their resource and/or funding body to enable full accreditation of the curation effort. The IntAct team is responsible for updating the data and producing a regular data release. The full IMEx dataset is made publicly available under an open Creative Commons Attribution 4.0 International licence (CC-BY4.0) as a single PSICQUIC service that can be accessed and searched via the IMEx Consortium website (ww.imexconsortium.org) and any other resource implementing the IMEx PSICQUIC webservice. These include the IntAct website (www.ebi.ac.uk/intact/) and the mentha web resource^22^, maintained by the MINT group.

Member databases may import some or all of the IMEx dataset back into their own resources. Partners may add other information to the IMEx dataset on their own websites or choose to only provide access to a subset of data that is of interest to their specific resource. For example, IID complements the IMEx data with interactions predicted by multiple machine learning and data mining algorithms, tissue and disease annotation context^23,24^, while MatrixDB only provides IMEx data pertaining to extracellular matrix proteins and glycosaminoglycans^25^. This model enables not only large interaction databases to contribute to the overall effort of IMEx but also allows annotation of molecular interactions by groups in data resources that do not maintain an interaction database (e.g. the UniProt Consortium). It also ensures that the dataset will continue to be maintained should funding be withdrawn or a resource decide to focus on a different area of research. For example, the data curated by the Microbial Protein Interaction Database (MPIDB), a former IMEx member, has been maintained and updated within the IntAct database since this resource ceased curation in 2013. The IntAct database additionally holds a considerable number of records curated by several of the IMEx Consortium members (primarily IntAct, MINT, DIP and UniProt), which pre-date the IMEx agreement. There is currently no concerted effort planned to bring this legacy data up to IMEx standards, although individual records may be re-curated on demand.

The Consortium benefits as a whole from the expertise of individual member databases. In addition to the extracellular matrix expertise of MatrixDB, HPIDB bring host-pathogen interaction experience and InnateDB focuses on the curation of molecular interactions involved in the innate immune system. The consortium structure enables a rapid response to new areas of biology, as demonstrated by the early release of a coronavirus interactome curated to full IMEx specifications based on pooled curation resources and the knowledge of individual members such as UniProtKB, which provided early access to protein sequence data^26^.

## The IMEx curation model

During the past 8 years, the IMEx curation model has been enriched and refined with new data types, methodologies and additional levels of detail. Every interaction curated by the IMEx Consortium reflects a piece of experimental evidence manually curated from a publication or directly submitted by a data producer. The IMEx Consortium adheres to a detailed curation model, which comprises all aspects of an interaction experiment. The IMEx record includes information on host organism (with details about the cell line or tissue in which the assay was performed), methods for interaction detection and participant identification, full details of the constructs (binding domains, effects of site-directed mutations, etc.), and further contextual information (e.g. any treatment of the host organism). All this information is mapped to CV terms, in particular those described by the HUPO PSI-MI CV (www.ebi.ac.uk/ols/ontologies/mi). Curated data records are linked to the source text from both figure legends and the main text body of the paper. This has enabled the use of these data to develop and assess deep learning approaches for text mining^27^.

The Consortium agreement^7^ was originally restricted to the curation of PPIs, but this proved limiting to some resources and compromised the ability of users to fully understand biological processes. Therefore, the remit of the Consortium has more recently extended to cover protein–protein complex, protein–small molecule, protein–carbohydrate, protein–nucleic acid and nucleic acid–nucleic acid interactions. Curation guidelines have been developed to enable the description of transcription factor-transcribed gene interactions. Furthermore, experimental techniques to capture ncRNA–protein and miRNA–mRNA interactions have been included in the CVs and curation guidelines. The Consortium is currently also examining the structured description of downstream effects of molecular interactions, such as the up- or down-regulation of gene expression.

Proteins are curated at the sequence level, using the UniProtKB database as the reference resource for proteins and peptides. The use of UniProtKB enables the curator, for each publication, to accurately describe the level of detail provided about the proteins and to use identifiers for the unambiguous annotation of each protein interactor. For instance, a publication may only give enough detail for an interactor to be mapped to any or all of the protein isoform products of a specific gene, or more specifically to a single protein isoform, or to a post-translationally cleaved peptide chain. UniProtKB supplies appropriate identifiers for all of these, and in each case supplies the corresponding underlying sequence. Binding regions can be aligned to known protein domains, as described by InterPro^28^. The effects of point mutations can be captured down to the amino acid level, using a CV to describe their effect on an interaction. To capture this level of detail, the use of a high-quality protein reference resource is essential. Reverse engineering protein to gene identifiers to enable network analysis of, for example, RNA-Seq data is a relatively trivial task but it is considerably more difficult, if not impossible, to map isoforms and binding domain data directly to a gene model or genomic sequence. Databases that curate PPI data directly to gene identifiers simply do not capture this wealth of information.

The detailed biocuration of binding domains, mutations^29^ and post-translational modifications (PTMs) requires that these coordinate-level mappings are kept synchronised with changes to the underlying protein sequence database. An update to a predictive gene model may result in a corresponding change to the protein sequence(s) derived from it. Interactions involving domains and/or residues of that protein sequence then require a corresponding update to ensure that the mapping to the updated sequence is correct. Update pipelines need to be run regularly, in line with the release cycle of the sequence database, namely every 8 weeks in the case of UniProtKB. This is a computationally complex set of procedures run at the EMBL-EBI on the entire IMEx content, ensuring its consistency and representing one advantage of maintaining this as a single dataset. Similarly, all CVs used to describe an aspect of an interaction are updated with every release.

Small molecule interactors, including carbohydrates and lipids, are mapped to the ChEBI database^30^, protein complexes to the Complex Portal^31^, mRNAs to Ensembl^32^ or ENA^33^, ncRNAs to RNAcentral^34^ and genes to Ensembl. Whilst in many cases the database representation of these entities is largely more stable than that of proteins, update pipelines for each of these will be enhanced and improved with time.

For analyses to be meaningful, data quality and full representation of experimental detail are of paramount importance. This is particularly required when working in an area of high data complexity and heterogeneity. However, capturing these data poses a significant challenge for curators who need to be familiar with an ever-growing set of experimental techniques. In order to minimise curation errors, all IMEx records are double-checked by a second curator prior to release. A more recent innovation is cross-database checking, which ensures that curation standards remain consistent between member databases. This is reinforced by Consortium workshops where rule changes and extensions are agreed, and joint curation exercises undertaken.

## IMEx data content and interactome coverage

The IMEx data is constantly growing, with a new data release approximately every 8 weeks. Whilst there are interactions captured for a wide range of species, Table 1 highlights the predominance of human PPI data. The significant fraction of human-other species interactions includes a considerable number of interactions tested with human proteins against close mammalian (primarily mouse) orthologues, but also host–pathogen interactions between human and viral/bacterial proteins. Other model organisms, such as *S. cerevisiae*, *E. coli* or *A. thaliana* are also well represented in the dataset and a curation focus for IMEx partners.

**Table 1:** Number of binary protein–protein interactions for selected model organisms present in the IMEx dataset, May 2020.

Binary pair	No. interactions IMEx/IMEx+legacy
Human–human (non-redundant)	490,061/521,353 (259,962/278,983)
Human–mouse	29,510/31,478
Human–any mammal^a^	526,772/561,062
Human–bacteria	10,720/10,800
Human–virus	21,480/22,811
Mouse–any mammal	51,578/80,230
Rat–any mammal	11,236/14,105
*Drosophila melanogaster*–*Drosophila melanogaster*	48,067/51,741
*Caenorhabditis elegans*–*Caenorhabditis elegans*	12,526/16,970
*Arabidopsis thaliana*–*Arabidopsis thaliana*	52,131/55,661
*Saccharomyces cerevisiae*–*Saccharomyces cerevisiae*	72,227/132,104
*Escherichia coli*–*Escherichia coli*	19,318/28,513

^a^Includes human–human.

The human IMEx data set is dominated by PPIs. The number of interactions involving other molecule types is considerably lower (Table 2), but is steadily increasing and will provide interesting extensions to biologically relevant networks.

**Table 2:** Number of binary molecular interactions for different interactor types present in the IMEx dataset, May 2020 (human–human data only).

Binary interaction	Interaction count
Protein–protein	490,061
Protein–protein complex^a^	155
Protein–small molecule	6469
Protein–DNA	7649
Protein–gene	1055
Protein–RNA (all types)	3511
RNA–RNA (all types)	490
miRNA–mRNA	121

^a^Protein complex refers to a stable macromolecular functional unit which can be linked to a corresponding entry in the Complex Portal. It is used by curators when the interactor identification can only be made at the complex level.

**Fig. 1:**
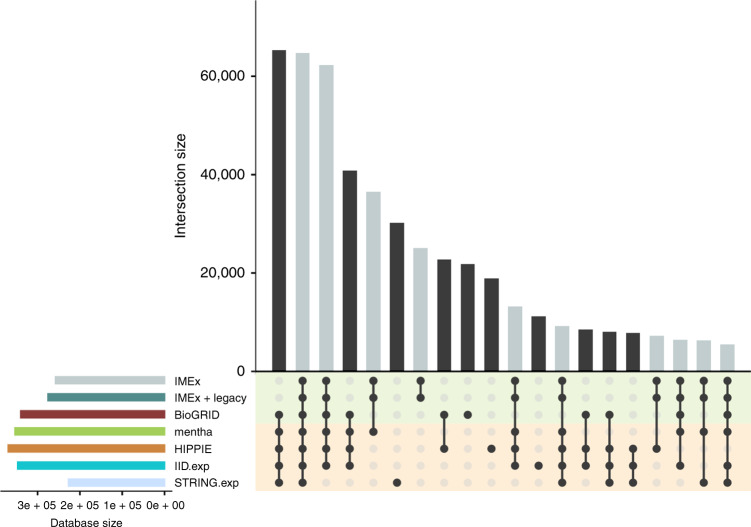
Overlap between Unique human PPIs from different resources. The intersection plot shows several PPI resources as well as legacy data curated by IMEx partners. Individual resource sizes are displayed as horizontal bars on the lower left corner of the image. Intersection sizes are shown as individual bars, with those including IMEx data highlighted in grey. Specific resources involved in each intersection are identified with connected solid black circles under the vertical bars, with unconnected circles representing pairs that are found exclusively in the corresponding database. Shading in the background of the circles helps differentiate between primary databases (green) and meta-databases (orange). Only the experimentally-derived content of the IID and STRING databases was used, excluding all predicted or text-mined interaction data. Only the 20 largest of all possible intersections are shown for clarity.

The curation performed by members of the IMEx Consortium represents a significant proportion of the interaction data found in publicly available databases. There is a degree of data redundancy with other databases that directly curate experimental data such as BioGRID^35^, and the IMEx dataset has been imported by meta-databases such as STRING^36^, mentha^22^, IID^14^ and HiPPIE^37^. Figure 1 shows the overlap between unique human interacting pairs in all these different resources, plus legacy data curated by IMEx partners as an intersection plot^38^. The greatest overlap exists between IMEx and BioGRID, which is well represented in the meta-databases considered, and between BioGRID and the rest of meta-databases. Figure 1 also highlights that the number of interacting pairs in BioGRID is larger than the IMEx dataset. This is due to the less detailed curation model adopted by BioGRID, which allows for faster curation and inclusion of data sets that are not considered as indicating physical interactions by the stringent IMEx rules (e.g., co-fractionation studies)^35^.

**Fig. 2:**
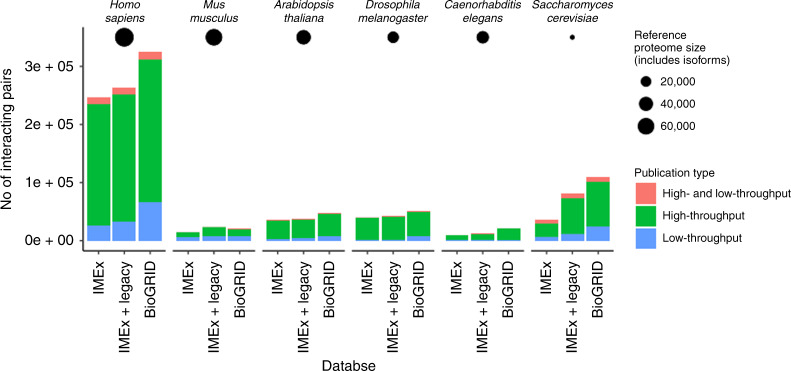
Number of unique interacting pairs in selected model organisms present in interaction databases. Differently coloured bars indicate the type of studies from which the data were curated. Data were accessed in January 2020.

Figure 2 compares the number of unique interacting pairs in the main model organisms (*Homo sapiens, Mus musculus*, *Arabidopsis thaliana, Drosophila melanogaster, Caenorhabditis elegans*, and *Saccharomyces cerevisiae*), highlighting also the type of studies from which the data was curated. Most of the interacting pairs hosted in primary databases come from high-throughput publications (>100 interacting pairs in an experiment or >100 interactors in an n-ary interaction). A comparison with BioGRID is included as this is the only other publicly available, manually curated interaction database.

**Fig. 3:**
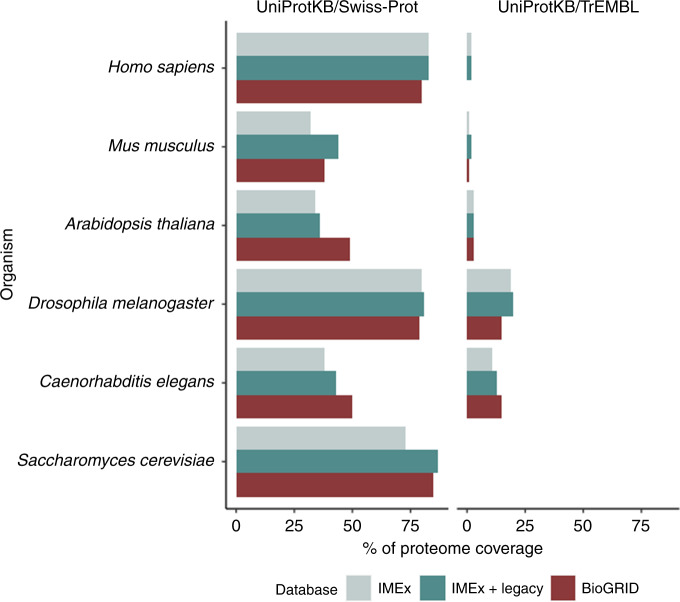
Coverage of UniProtKB model organism proteomes in interaction databases. Shown is the fraction of all proteome entries of key model organisms represented in UniProtKB/Swiss-Prot (manually reviewed) and UniProtKB/TrEMBL (computationally inferred) that are found in primary interaction databases (IMEx, IMEx + legacy data and BioGRID).

Finally, interactions in IMEx involve most of the representative/canonical proteins present in the human, mouse and *S. cerevisiae* proteomes as represented in the reviewed UniProtKB/Swiss-Prot (Fig. 3). Lower coverage is found in other model organisms such as *D. melanogaster* and *C. elegans*, partially because smaller fractions of these proteomes are represented in UniProtKB/Swiss-Prot. Many mappings are to proteins in the unreviewed UniProtKB/TrEMBL section and do not necessarily translate into UniProtKB/SwissProt when reviewed by UniProt curators. This makes the estimate of proteome coverage more difficult.

## Tools to visualize and query IMEx data

### ComplexViewer

The ComplexViewer^39^ has been designed specifically to visualise detailed data annotated by the IMEx Consortium. Its capabilities include a visual representation of a range of biomolecules as interactor types (proteins, small molecules and nucleic acids), interactions with more than two participants (n-ary interactions), sequence features relevant to the interaction (e.g., binding domains) and stoichiometry information. The data is taken from a JavaScript Object Notation format MI-JSON, which can be generated from any PSI-MI compliant data source using the Java Molecular Interactions library (JAMI, see below)^40^. This tool has been incorporated into the Complex Portal^31^, HumanMine^41^ (http://www.humanmine.org) and YeastMine^42^ (http://yeastmine.yeastgenome.org) data warehouses, and is freely available for implementation by additional resources (https://www.npmjs.com/package/complexviewer; http://biojs.io/d/complexviewer).

### ProtVista

The UniProt team has developed ProtVista^43^, an interactive tool for visualisation of a wide range of protein sequence features together in the same space. ProtVista is implemented using JavaScript and makes extensive use of D3 (https://d3js.org/), a library for producing dynamic, interactive data visualisations in web browsers. Work is currently underway to introduce an interaction track using the IMEx Consortium data, to visualise protein binding domains within the sequence of the protein represented in a UniProtKB entry, and to show the proteins to which it binds.

### PSICQUIC

PSICQUIC is a webservice created to enable computational access to standards-compliant molecular interaction data resources^20,21^. PSICQUIC defines a minimum set of standard SOAP (Simple Object Access Protocol) and REST (Representational state transfer) methods which accept a Molecular Interactions Query Language (MIQL) query as input and return molecular interaction information in one of the standard formats (PSI-XML2.5, MITAB). PSICQUIC enables the IMEx Consortium to make data available to the research community for rapid search and download. The current PSICQUIC implementation provides only limited access, however, to the wealth of data on molecule binding features, such as binding domains and the effect of site-directed mutations, and plans are in place to extend its capabilities.

### JAMI

The recently published JAMI library has been developed to import and export molecular interaction data in a variety of formats and versions, providing a single change-resilient software component^40^. Software and tools developed on top of the JAMI framework are able to integrate and support all versions of MITAB, PSI-MI XML, MI-JSON and XGMML. JAMI’s model interfaces are abstracted from the underlying format, masking the requirements of each data format from developers. JAMI is capable of serving the full richness of IMEx data to tools and resources built on this framework. It has been implemented by the IntAct and Complex Portal databases in addition to the InterMine data warehouse.

### RpsiXML

RpsiXML^44^ provides an R interface to PSI-MI XML2.5 files and can therefore be used to query the IMEx data. It provides a link between the detailed information held in these files and the diverse range of analytical tools provided in the Bioconductor software environment.

## Examples of uses of the IMEx dataset

### Building high-quality networks for data analysis

Protein interaction networks are used by biologists to understand the interconnectivity of signalling systems inside and outside of the cellular environments, exploring both physiologically normal and disease states. Network-based analysis is a powerful technique for extracting biological insights from large datasets. It enables researchers to associate proteins of unknown functions to human-curated pathways or identify clusters of interacting molecules, which may participate in the same biological process or belong to the same physical complex^45^. Network topology can suggest the biological properties and function of component molecules. Algorithms exist to analyse aspects of network structures (such as their scale-free, small world, geometric random or hierarchical nature), to calculate network metrics (e.g., centralities, shortest path, clustering coefficient and graphlets) or to investigate interaction motifs driving specific interactions between different biomolecules. The results of any network analysis will inevitably depend on the coverage and quality of the protein interaction network and the experimental conditions under which the interacting network was derived. IMEx data-derived networks have been incorporated into several clinical resources such as RD-Connect GPAP (https://platform.rd-connect.eu), DISNOR^46^, CancerGeneNET^47^ and OpenTargets^48^, enabling researchers to investigate genomic data and the consequence of disease variants.

The detailed IMEx curation model enables data selection and filtering on many levels and thus can contribute to the building of high-quality and context-specific networks. At the simplest level, such filtering can be performed using the manually curated ‘interaction type’ described by a series of terms contained in the MI CV. For example, it is possible to filter for ‘direct interaction’, or child terms, thus making specific smaller networks of the very highest quality. This approach was taken by Sacco et al.^49^ who queried IMEx data via the mentha interactome browser to build and analyse phosphatase sub-networks to identify new phosphatase substrates. Conversely, data types can be filtered out of a network. For example, computationally expanded data could be removed from a network using CV terms from the ‘curation content’ (MI:1045) branch which contains child terms such as ‘spoke expansion’ (MI:1060). IID supports filtering PPIs based on source, number of studies, number of bioassays, broad or detailed tissue, disease, cellular localisation and druggability. The MatrixDB resource supports filtering PPIs based on interaction detection method, gene expression and protein levels, disease, Gene Ontology terms, and UniProtKB keywords to build specific interactomes^25^, e.g. tissue-specific basement membrane interactomes, and to define consensus interactomes composed of the interactions common to all basement membranes^11^. Furthermore, it is possible to search the IntAct website or PSICQUIC webservice using the hierarchical structure of the ontology. For example, the term ‘lipid’ (CHEBI:18059) will identify all lipid-binding molecules even when the detailed annotation is to a cholesterol (CHEBI:16113) or phosphatidyl 3,4,5 inositol trisphosphate (CHEBI:16618) binding event.

Whilst these simple filters have been possible since the first release of IMEx curated data, for the last 6 years IMEx data have been scored using an implementation of MIscore^50^, thus enabling more sophisticated filtering. MIscore relies on the available annotation evidence associated with an interaction and represents the degree of confidence in the existence of a particular interaction. The scoring system takes three factors into account, and uses the CV terms added by the IMEx curators:

1. How the interaction was observed (interaction detection method; MI:0001)
2. The type of interaction: e.g., direct interaction, physical association and colocalization. (interaction type; MI:0190)
3. The number of publications reporting a specific interaction

The results are normalised on a 0–1 scale.

**Fig. 4:**
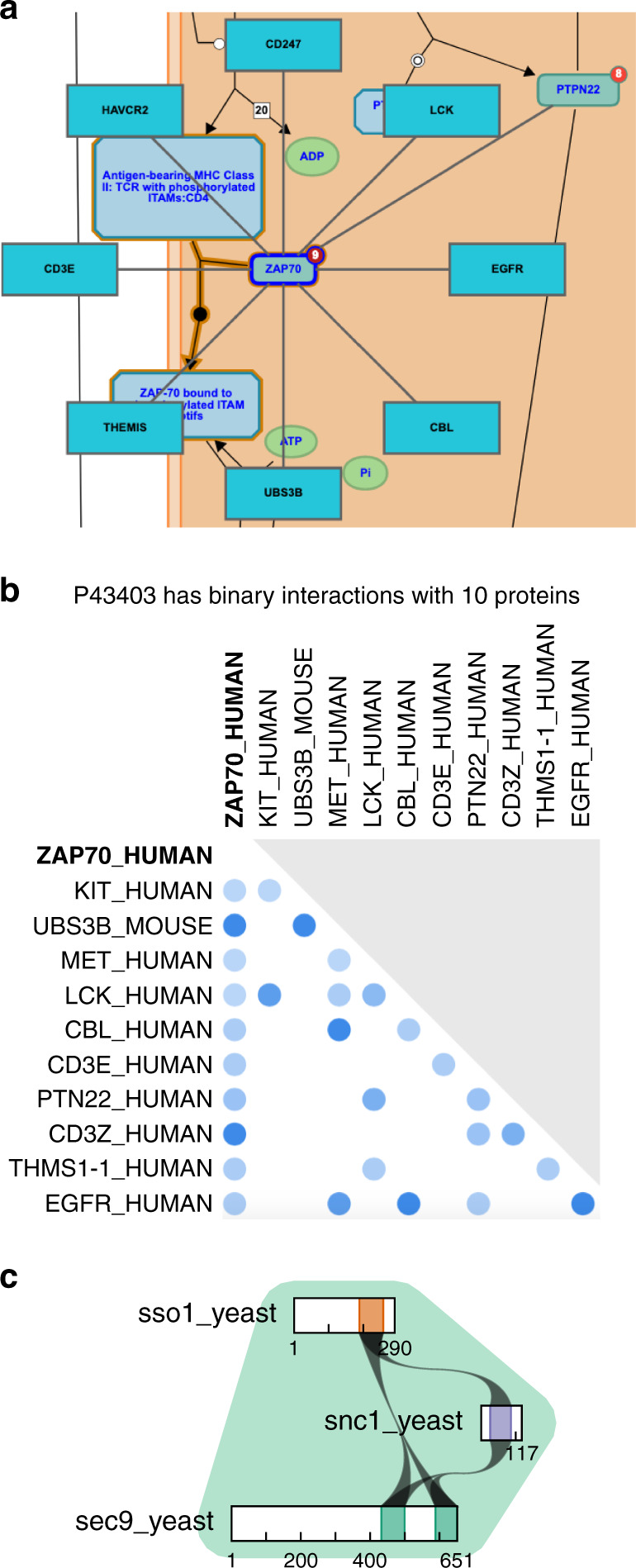
Presentation of IMEx data in different resources. **a**, **b** IMEx data for the human ZAP70 protein (P43403) represented in **a** Reactome and **b** UniProt. **c** IMEx-derived binding domain data for the *S. cerevisiae* vesicular SNARE complex SSO1-SEC9-SNC1 (CPX-1365) used in the Complex Portal.

Searching the IMEx dataset with the query “intact-miscore:[x TO y]” enables the user to select data subsets by confidence score. At the time of writing, the authors recommend a MIscore range of 0.45–1 to identify medium confidence interactions and 0.6–1 for high confidence sets. These thresholds approximately correspond to interactions found with at least two distinct pieces of evidence (MIscore > 0.45) or those found with three or more pieces of evidence, obtained with different methodologies (MIscore > 0.6). The MIscore functionality is used by the Reactome pathways database Molecular Interaction overlay^50,51^, which allows protein–protein or protein–small molecule interactions to be superimposed onto a pathway diagram. For example, Fig. 4a shows the tyrosine kinase ZAP70 (UniProtKB: P43403) in the Reactome TCR signaling pathway overlaid with 9 protein interactors imported from IMEx. The default setting provides fast access to a quarterly updated and locally hosted version of IMEx data with a MIscore threshold of 0.45, which selects ~50% of all interactions in IMEx. Interactions with a confidence level equal to or above this threshold will be visible in the viewport. The Reactome pathway analysis service also gives the user the option to set a more stringent MIscore filter using a slider feature, to select alternative interaction databases, and to perform an extended analysis that includes the IMEx interaction dataset served from the IntAct database.

Two studies independently applied data filtering to essentially the same network to investigate the biology of LRRK2, a protein linked to familial forms of Parkinson’s disease, using the data made publicly available by IMEx partners. Porras et al.^52^ filtered the dataset using MIscore to generate a high-confidence sub-network, which was used to produce a draft list of high confidence LRRK2 interacting partners. Manzoni et al.^53^ filtered by the number of publications reporting an interaction and then performed Gene Ontology network analysis on the LRRK2 interactome with edges identified by 2 or more publications. Both groups showed that the LRRK2 network was associated with terms referring to transport, cellular organization, vesicles and the cytoskeleton. Experimental data have since shown LRRK2 to be associated with selected Rab GTPases, and are now also present in the IMEx dataset^54^.

MIscore was designed to be customisable and a different version of the algorithm is used to identify binary interactions for export to the UniProtKB Interaction lines and to the Gene Ontology database. All binary interaction evidence in the IntAct database, including the data generated by spoke expansion of co-complex data, are clustered to produce a non-redundant set of protein pairs. Each binary pair is then scored using a variation of MIscore to give a simple addition of the accumulated value of a weighted score for the interaction detection method and the interaction type for each interaction evidence associated with that binary pair. Once the interactions have been scored, a threshold is established, below which the interaction is not exported to UniProtKB and to the Gene Ontology annotation files. The threshold is stringent enough to ensure no interaction is exported based on only a single piece of experimental evidence. Additional rules ensure that any protein pair scoring above the cut-off must also include at least one piece of evidence of a binary pair, excluding spoke expanded and co-localisation data, to be exported to UniProtKB and Gene Ontology annotation. The UniProtKB dataset is then displayed in the appropriate entries in an adjacency viewer (Fig. 4b).

The IMEx dataset has also been used as a gold-standard training set in text-mining exercises such as the Biocreative competitions. These exercises make use of IMEx data partly because a linkage between figures and experiments has been maintained during the process of IMEx biocuration^55^. Furthermore, in a subset of IMEx entries, sentences identifying interacting entities and the interaction detection method have been systematically captured in the ‘source-text’ annotation field. These sentences are highlighted for readers on abstracts and full text articles in Europe PMC via the SciLite tool^56^.

### Characterising protein isoforms and features

Most eukaryotic protein-coding genes transcribe more than one isoform. The different functions of isoforms are sometimes known or can be inferred (for example specific isoforms do/do not contain certain functional domains), but in many cases the biological significance of multiple isoforms derived from the same gene is not understood. However, the different interaction patterns of associated isoforms may provide an indication of their different biological functions by analysing their respective binding partners. In 2013, Talavera et al.^57^ published an editorial stating that “it is crucial to the advance of basic and medical research that interactions are reported on an isoform-to-isoform basis and that databases switch to a similar approach”. The IMEx databases curate this information, whenever the data is made available by authors, making isoform comparisons possible. UniProtKB identifiers enable curators to differentiate between transcripts being identified at the isoform or canonical (reference sequence) level. Over 100,000 interactions in IMEx (~12% of IMEx data) contain specific isoform information, with more than 11,000 records containing specific isoform–isoform interactions. The UniProtKB database recently (release 2020_02) refactored the Interaction section of their records to improve the display of isoform data imported from IMEx. It is anticipated that the availability of such data will increase as protein identification techniques improve or as authors realise the value of such data and include this level of detail in publications.

IMEx also captures so-called negative interactions, which will be of increasing use in the future. These data largely pertain to isoform-specific interactors, and describe cases where certain isoforms of a gene bind to a bait protein, while other isoforms of the same gene do not bind to the same bait in the same assay system. IMEx curation rules mandate publication of the protein expression levels of the negative interactors to exclude poor protein expression as a reason for the lack of interaction.

To fully comprehend protein interactions, researchers frequently need to identify the sequence region to which a molecule binds and any modification to that sequence. Any change to an amino acid sequence has the potential to influence the molecules with which the protein interacts. The IMEx Consortium captures these variations, thereby supporting the analysis of their downstream effects as shown in the examples below.

### Binding domains

One critical piece of information captured by the information-rich IMEx curation model is the minimum ‘sufficient binding region’ (MI:0442) or ‘necessary binding region’ (MI:0429) of a protein derived from an interaction experiment. When a binding domain maps to a known protein domain, a cross-reference to the appropriate InterPro entry is added. Capture of data to this level of detail has enabled, for example, an improved understanding of the role of the SH2 domain including a classification of its target protein specificity^58^ and the identification of the WD40 domain as potentially being directly involved in ncRNA interactions^59^. The binding regions captured by the IMEx Consortium have also been used for the precise mapping of binding domains within protein complexes in the EBI Complex Portal^31^, e.g. in the *Saccharomyces cerevisiae* vesicular SNARE complex SSO1-SEC9-SNC1 (CPX-1365) formed by SNARE-SNARE domain binding (Fig. 4c).

### Post-translational modifications

PTMs, such as the phosphorylation of amino acid side chains, increase the complexity of the proteome and are essential for driving molecular interactions. These modifications can change PPIs by causing protein oligomerization and aggregation, binding to or dissociation from other proteins, protein conformational changes or local unfolding. The IMEx Consortium differentiates between a ‘prerequisite-ptm’ (MI:0638), which is required for an interaction to occur, and an ‘observed-ptm’ (MI:0925), which has been experimentally validated but not shown to be required for the interaction. For example, a phosphorylation event can introduce a charge in a hydrophobic environment, destabilising an interaction. This can be systematically described using CV terms such as ‘ptm disrupting an interaction’ (MI:1225).

Post-translational cleavage of a polypeptide is also a PTM yielding a mature protein or bioactive peptide chains, which may have interaction repertoires very different from those of the originating full-length transcript. Using the mature protein chain identifiers supplied by the UniProtKB database allows IMEx curators to accurately capture the form of the protein used in the assay. This is of particular importance for the annotation of the interactions of viral proteins where one gene in the viral genome may encode multiple proteins. The protein interactions of these post-processed protein and peptide chains cannot be meaningfully described when protein interactions are only captured at the gene level.

Reversible and transient PTMs transmit and amplify signals in a highly regulated manner by reversible site-specific modulation, and thus play a key role in signal transduction^60^. PTMs are often the result of an enzyme acting on a substrate and the enzyme–substrate reaction can be taken as evidence of a direct interaction in the IMEx data model. The PTM resulting from this interaction is additionally captured, using the ‘resulting-PTM’ (MI:0639) term. Cell signalling resources, such as the SIGNOR database^61^ have used the relationships between enzymes and substrates from the IMEx dataset and the effects of resulting PTMs on interactions to derive causal interactions.

More recently, the IMEx Consortium has also started to capture the effects of chemical modifications of RNA molecules, several of which undergo specific nucleotide modifications during their maturation.

### Point mutations

To understand how amino acid variations influence protein function and stability, researchers have for many years examined the effect of induced point mutations on protein interactions. These targeted changes to the amino acid sequence of a protein may mimic known sequence variants, remove post-translational modification sites, disrupt regions required for protein stability or alter the properties of protein binding domains. The IMEx Consortium has been collecting these data^29^ using CV terms to describe the observed effects such as ‘mutation decreasing interaction strength’ (MI:0116), ‘mutation increasing interaction rate’ (MI:1131) or ‘mutation causing an interaction’ (MI:2227). The curation rules have recently been extended to include deep mutational scanning data such as described by Woodsmith et al.^62^. At the time of writing, this set consists of 58,000 point mutations representing 20,000 interaction evidences annotated with differentially reported effects. To make these data more readily available to the user community, the IMEx Consortium has recently concatenated this dataset and made it available in a tab-delimited format (FeatureTAB)^29^. This new data format includes details of the position and the amino acid change of the mutation, the interacting molecules and the effect of the mutation on the interaction.

**Fig. 5:**
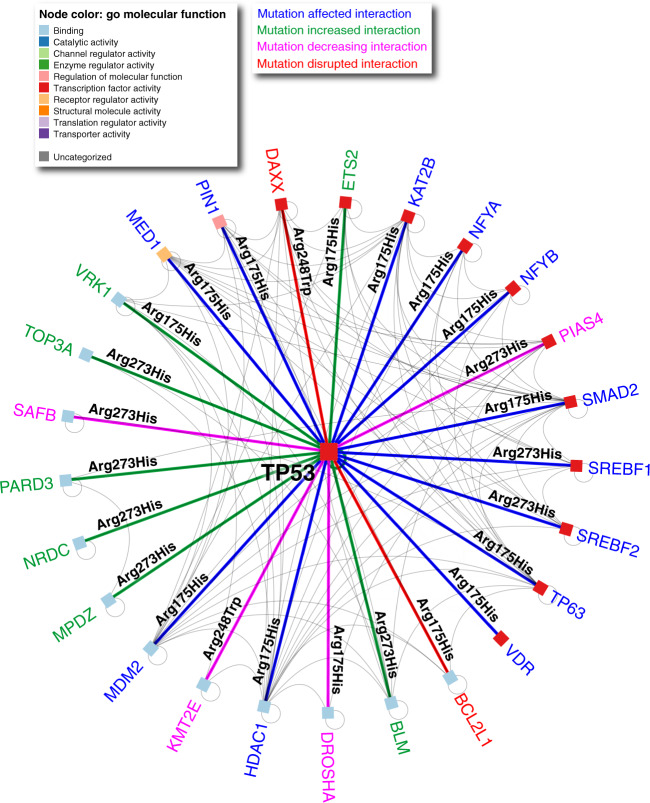
Mutation-specific TP53 interaction network. Survival-related patient mutation data^57^ overlaid onto the mutation-specific TP53 network derived from interactions from IID (version 2020-05) and mutation-specific interaction data from IMEx. Survival-related mutations are shown on the network edges. It should be noted that many interactions are affected by many more mutations, but these are not necessarily linked to ovarian cancer. The network is visualised in NAViGaTOR^73^ ver 3.0.13. Node colour corresponds to GO molecular function, edge colour represents the effect of the mutation on the interaction.

The IMEx Consortium mutation-specific dataset is available to download at http://ftp.ebi.ac.uk/pub/databases/intact/current/various/mutations.tsv. The data have already been used to provide potential mechanisms of action for disease related amino acid variants, to investigate the destabilising effect of mutations on structural models of protein–protein interfaces, and to benchmark tools predicting the effect of SNPs on protein function^29^. In another recent study, both the protein interaction network from IID^23^ and the mutation-specific interaction dataset have been used to explain survival and treatment resistance in ovarian cancer^63^. Patients with a TP53 p.Arg175His mutation have a poor prognosis, which may be explained by chemoresistance due to a disrupted TP53 interaction with the BCL2 complex^64,65^ as well as several additional mechanisms, as highlighted in Fig. 5.

The IMEx database also collects information concerning the nucleotides involved in miRNA-mRNA interactions and the effect of nucleic acid substitutions on binding. These data are accurately mapped on the mRNA sequence, making them a valuable resource for modelling miRNA interactions and the regulation of gene expression at specific targets. To the best of our knowledge, the IMEx Consortium is the only group capturing this information.

### Protein tags

Protein tags are chemical additions to a molecule to enable its identification and/or purification. They often consist of peptide sequences genetically attached onto a recombinant protein. As the nature and position of a tag may affect its interaction profile, this information must be recorded to enable researchers to fully comprehend the consequences of such changes^66^. For example, a tag expressed as either an N- or C-terminal fusion of Ebola Virus VP24 protein identified 48 and 51 interacting human proteins, respectively, of which only 40 proteins are common to both fusions^67,68^.

### Contextualising protein interactions with metadata

As detailed above, the interaction repertoire of a protein depends on many factors, not least the cellular environment in which they are expressed. The detailed metadata now captured in an IMEx record have been significantly expanded since the work of the Consortium was first described. Most techniques for measuring interactions do not use native conditions and, when described by the author in the publication, these data are captured in a systematic way using ontologies and CV terms. In addition to full details on the host organism, experimental methodology and construct details, IMEx curators now routinely capture expression level, any full or partial purification of a molecule, and the method by which constructs are delivered or engineered into a cell or expression system. This information allows analysing the effects of environmental change on interaction patterns. For example, it has been noted that the Huh7.5 cell line differs from the 7.0 parent cells by a single mutation (p.Thr55Ile) in Retinoic acid-inducible gene I (RIG-I) protein, which impairs interferon signaling^69^. Analyses by the MacCarthy group using data uniquely provided by IMEx suggest notable differences in the pattern of host proteins interacting with HCV proteins from these two different cell lines^67^. Huh7 proteins interacting with proteins from HCV are involved in cancer, apoptosis, immune defence response and cell cycle functions, whilst the equivalent Huh7.5 proteins are enriched for protein folding, localisation, and transport.

Recent changes to both the database model and PSI-MI XML3.0 download format^18^ allow curators to capture dynamic interactions, such as changes in sub-network composition at different stages of the cell cycle or in response to changing concentrations of an agonist/antagonist, pH changes and other factors. It is also possible to describe the directionality of a reaction or binding event and the result of a directional binding event, such as an up- or down-regulation of the target’s activity. Once sufficient data have been accumulated, they will be made available to users in a new tab-delimited format^70^.

## Future and sustainability

Since first described in 2012, the IMEx Consortium has gained new member data resources and curation groups; these include the UniProt Consortium and the Functional Gene Annotation group at University College London. Some previous member resources ― such as MPIDB ― are no longer in existence, but the data remain in the IntAct database and are updated with each release of UniProt. The IMEx Consortium has released almost 900,000 interaction evidences to IMEx curation standards and continues to provide access to another 100,000 legacy binary evidences. It has expanded the IMEx dataset to include new interactor types, new methodologies and new data types such as dynamic interactions. The Consortium remains open to the participation of new partners, and will make access to the IntAct editorial tool, curation training and data quality control available on request. For detailed information on both IMEx membership and on data deposition, please see https://www.imexconsortium.org.

Data producers can contribute to the IMEx project in three key ways. First, by depositing interaction data with one of the Consortium partners as an integral part of the publication process. Second, by always clearly identifying all the constructs used in any interaction experiment^71^, ideally by the addition of an accession number from a database such as UniProtKB or by making the species of origin of any clone very clear (for example “Human hemagglutinin (HA)-tagged RRP1B (Q14684)….”). Additional sequence detail will enable mapping to the correct isoform, when relevant. Third, data producers can request the curation of papers, particularly when these supply interactions missing from an interactome, or bring additional details to an interaction that is not already present in the dataset. Researchers seeking assistance with these requirements may contact intact-help@ebi.ac.uk.

The IMEx Consortium received EC funding to establish itself and has more recently received UK BBSRC and NIH grants. Currently, the consortium relies on localised, national funding and research grants to maintain resources. IMEx is the first Consortium to be recognised as an ELIXIR core resource, highlighting the importance this organisation places on database collaboration and data sharing. ELIXIR has supplied local funding to support member databases and to fund Consortia meetings. It is hoped that the recognition by ELIXIR will result in longer term funding for Consortium-wide activities. The Global Biodata Coalition^72^ is currently looking to extend this model of sustainable funding for core-data resources in the life sciences giving hope for the long-term future of key resources such as the IMEx Consortium.
